# Biodegradable Green Composites: Effects of Potassium Permanganate (KMnO_4_) Treatment on Thermal, Mechanical, and Morphological Behavior of Butea Parviflora (BP) Fibers

**DOI:** 10.3390/polym15092197

**Published:** 2023-05-05

**Authors:** M. Abisha, R. Krishna Priya, Krishna Prakash Arunachalam, Siva Avudaiappan, Erick I. Saavedra Flores, Pablo Fernando Parra

**Affiliations:** 1 PG & Research Department of Physics, Holy Cross College, Nagercoil, Affiliated to Manonmaniam Sundaranar University, Tirunelveli 627012, India; 2 Department of Civil Engineering, University College of Engineering Nagercoil, Anna University, Nagercoil 629004, India; krishnaprakash3191@gmail.com; 3 Departamento de Ingeniería Civil, Universidad de Concepción, Concepción 4070386, Chile; 4 Centro Nacional de Excelencia para la Industria de la Madera (CENAMAD), Pontificia Universidad Católica de Chile, Av. Vicuña Mackenna 4860, Santiago 8330024, Chile; 5 Department of Physiology, Saveetha Dental College and Hospitals, SIMATS, Chennai 600077, India; 6 Departamento de Ingeniería en Obras Civiles, Universidad de Santiago de Chile, Av. Ecuador 3659, Estación Central, Santiago 8320000, Chile; 7 Faculty of Engineering and Sciences, Universidad Adolfo Ibáñez, Santiago 7941169, Chile

**Keywords:** cellulosic fiber, crystallinity, sustainability, green composites

## Abstract

This study emphasizes the importance of utilizing biodegradable material Butea parviflora (BP) fiber for sustainable solutions. BP fiber offers numerous ecological benefits, such as being lightweight, biodegradable, and affordable to recycle. The study examines the effects of potassium permanganate (KMnO_4_) treatment on BP fiber and analyzes its physical and chemical behavior using various methods, including X-ray Diffraction (XRD) analysis, tensile testing, thermogravimetric analysis, thermal conductivity, Scanning Electron Microscopy (SEM), and Fourier Transform Infrared spectroscopic (FTIR) analysis. The results demonstrate that BP fiber possesses low density (1.40 g/cc) and high cellulose content (59.4%), which fosters compatibility between the matrix and resin. XRD analysis indicates a high crystallinity index (83.47%) and crystallite size (6.4 nm), showcasing exceptional crystalline behavior. Treated fibers exhibit improved tensile strength (198 MPa) and Young’s modulus (4.40 GPa) compared to untreated fibers (tensile strength—92 MPa, tensile modulus—2.16 GPa). The Tg-DTA thermograms reveal the fiber’s thermal resistance up to 240 °C with a kinetic activation energy between 62.80–63.46 KJ/mol. Additionally, the lowered thermal conductivity (K) from Lee’s disc experiment suggests that BP fiber could be used in insulation applications. SEM photographic results display effective surface roughness for composite making, and FTIR studies reveal vibrational variations of cellulosic functional groups, which correlates with increased cellulosic behavior. Overall, the study affirms the potential of BP fiber as a reinforcing material for composite-making while emphasizing the importance of utilizing biodegradable materials for sustainability.

## 1. Introduction

Technological advancement provides valuable resources along with ruins that are primarily non-biodegradable. There are high hopes that bio composites infused with natural fibers will attain a pollution-free environment and reduce synthetic acquisition. Reduced mass fraction and density are the two key variables that prioritize the use of natural fibers as reinforcements. A key factor that impacts the utility and availability of product in the market is its structural design, which is predominantly overtaken by synthetic materials. Now, the natural fiber composites are turning out to be trend-setters with their adaptability to deform to various designer structures. Plant fibers exhibit a semicrystalline behavior. Plant fibers offer numerous benefits over synthetic fibers, including being renewable, biodegradable, and compostable. In addition, plant fibers require less energy, money, and chemicals to produce, making them an environmentally friendly option for both consumers and businesses. Natural fibers, such as *hemp*, *sisal*, *flax*, and *musa fibers*, have been extensively used in various applications, including automotive parts, industrial usage, and acoustic applications [1]. Bark fibers, such as *kenaf fibers*, are commonly used in paper-making, construction, and vehicle parts [2]. Enzymatically retted flax is highly sought-after for linen production, while *sisal fibers* are well-regarded for their use in wiring and civil applications [1]. However, green fibers have some areas of concern, such as hydrophilicity, lower processing temperatures, and lower durability compared to synthetic fibers, which can be overcome with various treatment processes, including alkalinization, bleaching, benzoylation, acetylation, and silane treatment [3,4]. Alkali treatment, which breaks down alkali-sensitive OH groups, is the preferred method, as it removes amorphous constituents and oils from the fibers, leading to improved mechanical properties [5]. The addition of permanganate treatment to alkali treatment improves the water repellency, crystallinity, and mechanical behavior of the fibers, although extended soaking in high concentrations of both NaOH and KMnO_4_ can lead to the removal of fiber components and subsequent reduction in tensile strength, as fiber bundles begin to separate into individual fibrils. Effective coupling between fibers and the matrix in composites is crucial for establishing an interfacial bond and transferring stress effectively. The use of silane coupling agents after alkali treatment has been shown to improve the flexural and tensile properties of jute and kenaf fiber composites [6]. Benzoyl chloride treatment, pretreated with NaOH, has been found to be effective in improving the tensile and hydrophobic behavior of *areca sheath fibers*, *palmyra palm leaf stalk fibers*, and *kenaf core powder* composites, as the benzoyl group displaces OH groups in the fibers, providing better hydrophobic properties. However, fibers must be carefully scrutinized during chemical treatment to avoid excessive exposure to chemicals, which can diminish their usefulness before being used in composites [7].

Fibers must be pulled out correctly with lowered amorphous entity before considering them for any reinforcements [8]. Plant fiber that is being used as reinforcement determines the entire behavior of composites. Unlike synthetic fibers, the strength of green fibers is largely linked to the climatic conditions, soil nature, maturity, preservation time, processing methods of fibers, etc. [9]. Fiber-resin adhesion is achieved after chemical modification, which helps to rise interfacial energy, thereby enhancing thermal and mechanical ability of composites [1]. Fiber orientation, fiber dispersion, matrix selection, and interfacial bonding are the prime traits approached in making composites. Floor trays, door trim panels, and utility areas inside the automobiles are a few outcomes of fiber composites from *palm*, *flax*, and *kenaf* [10,11]. Replacing wooden laminates with fiber-reinforced composites is possible because of their mechanical strength [12]. Green composites also are attractive for wall insulation and floor lamination structural utilities. However, the hunt for new green composites would replace non-renewable resources and improve pollution management [11,13].

The present work throws a light on the potential of Butea parviflora (BP) fiber in polymer applications. BP fiber is commonly found in Southeast Asia, especially in India, Thailand, and Indonesia. Fibers are obtained from the inner bark stem of this woody climber. The length of the fibers can vary from a few centimeters to several meters, and their color can range from light to dark brown. Butea parviflora is one of many plants in the genus “Butea” that is prized for its possible medicinal, anti-microbial, and pharmacological properties [14]. Bark fibers are utilized in making cordage due to their outperforming strength [15,16]. Fiber length up to 1 m can be collected from the Butea plant and could be an advantage for making unidirectional and bidirectional composites.

Butea parviflora (BP) fiber is an eco-friendly and sustainable fiber option because it is biodegradable, renewable, and requires minimal processing. However, there is limited research available on its physical and mechanical properties, as well as its potential uses in various industries. KMnO_4_ action on fibers could improve the tensile strength while retarding moisture absorption [17]. Characterization tests such as X-ray diffraction, physiochemical analysis, mechanical testing, thermogravimetric and thermal conductivity studies, SEM examination, and FTIR functional group analysis gave a wide affirmation for accepting BP fiber as reinforcement for composite applications.

The innovation of this study lies in the utilization of Butea parviflora (BP) fiber as a sustainable and biodegradable material for composite making. The study showcases the exceptional properties of BP fiber, including its high cellulose content, low density, and high crystallinity index, which make it a suitable reinforcing material for composites. Additionally, the study examines the effects of potassium permanganate treatment on BP fiber, which enhances its tensile strength and Young’s modulus, improves its thermal stability, and lowers its thermal conductivity, making it suitable for insulation applications. The findings of this study open up new avenues for utilizing BP fiber as a sustainable and environmentally friendly material in the composite industry. Moreover, the study highlights the importance of utilizing biodegradable materials for sustainability, which is essential for promoting a greener and cleaner environment. Overall, the innovation of this study lies in the potential of BP fiber as a sustainable and eco-friendly alternative to conventional reinforcing materials, which could help in reducing the carbon footprint of the composite industry.

## 2. Methodologies

### 2.1. Fiber Extraction and Treatment

**Figure 1:**
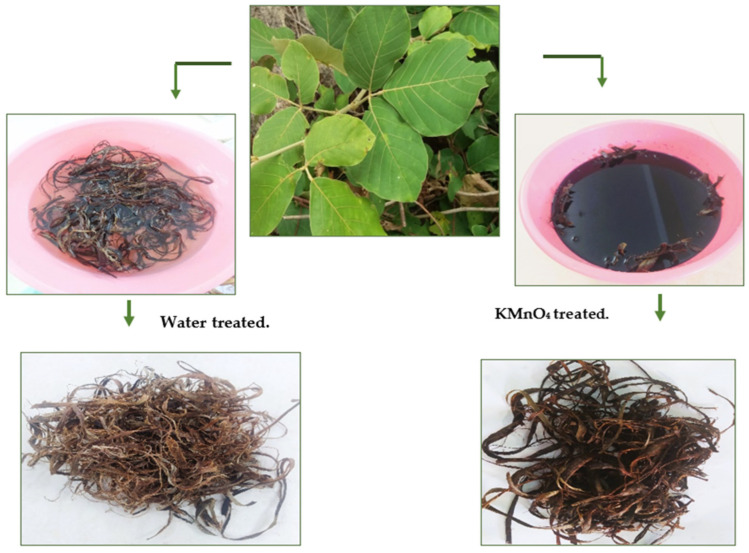
Fibers from Butea parviflora (BP) plant.

Butea parviflora (BP) is a woody climber belonging to the Fabaceae family. It is found in the regions of Kanniyakumari, Tamil Nadu. The whole plant is fibrous, but the strength and texture vary from the outer to inner space. Due to its strength, bark fibers of Butea plant are used by locals for tying, knotting, and binding requirements. Bark contains 1% rotenone, which is used as an insecticide. In order to separate mature fibers from the plant, a metal teeth tool is used. Collected fibers are treated in distilled water and potassium permanganate; treatment involves soaking of fibers in 0.1 M NaOH solution for 20 min, followed by 15 min in the same molarity of potassium permanganate at 27 °C. Treated fibers are rinsed in distilled water. Fibers are first drained in shade, then dried completely using a vacuum desiccator. Variation in soaking time and concentration of KMnO_4_ will bring varying results. Different treatments at 0.025% and 0.05% of KMnO_4_ on the Musa plant eliminated the non-cellulosic debris and manifested the crystalline and tensile property of fiber. The delignification process that takes place during permanganate action facilitates the rearrangement of microfibrils, resulting in an improvement in crystallinity. Moreover, the wetting of fibers in 0.1% KMnO_4_ induces capillary forces that increase their resistance to water [18]. Figure 1. presents water and KMnO_4_ treated BP fibers.

### 2.2. Physical Properties

The purpose of chemical procedures is to strengthen the docking of fibers in the composite [7], which has a direct influence on the physical properties as well. Moisture enters through the amorphous region and gets arrested within the microfibrils. NaOH and KMnO_4_ treatment on fibers will prevent the intrusion of moisture into the core of the composites; thus, they can endure load and delay the origin of fracture [11,13]. An average of 30 fiber strands are examined and their average values are taken for finding their physical properties. Physical outcomes of Butea fibers are compared with other fibers in Table 1.

#### 2.2.1. Diameter and Aspect Ratios of BP

Diameter and length of BP is observed through an optical microscope and ruler. Modulus of fibers increases with decrease in diameter. Additionally, the fiber’s diameter varies with different plant layers. Permanganate treatment on BP fibers reduced the diameter, which would favor a compactivity with the matrix for composite making [19].

High fiber aspect ratios could accelerate the strength of composites [20]. Aspect ratios of permanganate-treated fiber (305) is greater than untreated BP (175). All readings were computed under normal room temperature and pressure.

#### 2.2.2. Linear Density of BP

The fiber’s fineness is expressed as mass per unit length since diameter and cross-sectional shape are not even along the length. Tensile properties of fiber increases with linear density. Yarned ramie fiber showed improved tensile value, while the linear density was 65 tex [21,22,23]. Linear density is found using,

(1) $$\mathrm{Linear}\;\mathrm{density}\;(\mathrm{LD})=\frac{\mathrm{mass}\;\mathrm{of}\;\mathrm{fibers}\;(\mathrm{grams})}{\mathrm{length}\;\mathrm{of}\;\mathrm{fibers}\;(\mathrm{meter})}$$

LD of Butea fibers is higher than *Coccinia grandis*. Aspect ratio has a direct influence on the better mechanical behavior of the physical traits of fiber. Failure of composites happens at a slower rate when the reinforced fiber has brilliant length-to-diameter ratios [20]. Approximate length taken for analysis is 10 cm.

#### 2.2.3. Density of BP Using Pycnometer

Commercialization of natural fiber-based products over synthetic ones is primarily based on lowered density. Density of carbon (1.8 g/cc) and aramid fibers (1.45 g/cc) are higher than many plant fibers [24]. Alkaline treatment increases the density of fibers by removing less dense non-cellulosic components such as hemicelluloses and lignin [25]. Chemical modification brings a change by lowering the crystal defect and distortion that result in bulk density of fibers [26]. Increased density values in the permanganate-treated Butea fiber satisfy the above reasons. Using the liquid pycnometer method, density was found by inserting toluene as the immersion liquid [27,28,29]. Density is derived from,

(2) $$\rho f=\frac{\left(m_{b}-m_{a}\right)}{\left[(m_{c}-m_{a})-(m_{d}-m_{b}\right)}\rho t$$

where *m_a_*—empty pycnometer mass;

*m_b_*—mass of (pycnometer + chopped fibers);

*m_c_*—mass of (toluene + pycnometer);

*m_d_*—mass of (toluene + pycnometer + chopped fibers).

**Table 1:** Physical outcomes of treated Butea parviflora (BP) with other fibers.

Fibers	Diameter	Aspect Ratio (L/D)	Linear Density (tex)	Density (g/cc)	References
Raw BP	500 µm	174.59	311.86	1.238	Present work
KMnO_4_-treated BP	488 µm	305.00	459.00	1.409	Present work
*Ariel roots of banyan fiber*	0.09—0.14 µm	–	–	1.23	[29]
*Cissus quadrangularis*	130 µm	–	–	1.29	[30]
*Acacia leucophloea*	168.5 µm	–	–	1.385	[31]
*Coccinia grandis*	543—621 µm	–	130.90	1.517	[32]

## 3. Characterization Studies

### 3.1. XRD Studies

X-ray diffraction is an effective way of exposing the arrangement of atoms, although the main compartments of plants are cellulose and hemicellulose. The analysis was carried out using the Bruker AXS–D8 Advance Model diffractometer, running at 40 kV voltage; 2θ values were taken between 0° and 70°. Crystallinity index of BP fiber was analyzed from Segal empirical formula [33]

(3) $${\mathrm{CI}}_{\mathrm{BP}}=\frac{I_{cry—I_{am}}}{I_{cry}}\times 100\%$$

where I_cry_—intensity at (002) plane, I_am_—intensity at (110) plane. Using Scherrer’s equation, crystallite size is computed [34,35]

(4) $$\mathrm{CS}=\frac{K\lambda }{\beta_{200}cos\theta }$$

where K—Scherrer’s constant, λ—0.154 nm, β_200_—full width half maximum around 23°, θ—Bragg’s angle.

### 3.2. Single Fiber Tensile Test

Strength of BP along with elongation data from the (Zwick/Roell) instrument are used to determine the tensile modulus of KMnO_4_-treated BP fibers. A relative humidity and temperature of 65% and 21 °C was maintained throughout the experiments. The testing procedure fixed the gauge length to be 50 mm to obtain a transverse rate of 30 mm/min. The microfibrillar angle (α), which plays a vital part in the mechanical output, is obtained from the global deformation equation [30].

(5) $$ԑ=ln(1+\frac{\Delta L}{L})=-ln(\cos\alpha )$$

where ε—strain, α—MFA, L—BP fiber’s length, and ΔL—change in length.

### 3.3. Thermogravimetric Studies

Enhanced thermal characteristics of natural fibers would extend the useful life of composite materials reinforced with plant fibers. The mass loss and enthalpy changes were investigated using the Perkin Elmer STA 6000 Model at STIC, Cochin, from the manufacturer Perkin Elmer Inc, Mumbai, India. The analysis is noted between 40 to 800 °C, with a heating rate of 20 °C for 60 s. Kinetic activation energy was evaluated from the DTG data using Coats–Redfern approximation. Differential scanning calorimetric values are obtained from the same instrument.

### 3.4. Thermal Conductivity Studies

Thermal responsiveness is a key factor in assessing the insulating utilities. With the use of Lee’s disc arrangement, the K value of biofibers is calculated. After the temperature has stabilized, the disc setup is left to cool to mark the subsequent temperature decline. K value was determined using the following equation [36]

(6) k=mxdr+2hπr^2T1−T22r+2hdT/dt W/m/K

where m—Lee’s disc’s mass, d—specimen thickness, x—specific heat, r, h—radius and Lee’s disc’s thickness, dT/dt—slope of temperature rate, T1—steady temperature and T2—temperature of Lee’s disc.

### 3.5. Morphological Studies Using SEM

SEM examinations are used to inspect the flaws and smoothness on the fiber morphology. JEOL 6390LA/OXFORD XMXN, from JEOL India PVT LTD; South Delhi, a subsidiary company of JEOL Limited, Japan. It used to scan the surface configuration of fibers, with an accelerating voltage ranging from 0.5 to 30 kV. 

### 3.6. FTIR Analysis

Vibrational assignments linked to the biofiber for the variable functional groups are emphasized using Fourier-transform infrared spectroscopy. With the instrumental support of FTIR-8400S spectrum from the manufacturer Shimadzu in Japan, using KBr matrix and a speed of 45 per minute, vibrational variations amongst the cellulosic groups of BP fibers are registered.

## 4. Results and Discussion

### 4.1. Chemical Composition

The cellular components cellulose, hemicellulose, and lignin are mostly resonated with the age of plants, the source of the fiber section, and the extraction pattern [37]. Standard analytical methods were used to determine the chemical composition of each fiber sample. The acid detergent method was used to measure cellulose content, while hemicellulose content was determined using the neutral detergent method. The Klason method was used to determine lignin content, and moisture content was measured by drying the samples at 105 °C until a constant weight was obtained. Soxhlet extraction with hexane as the solvent was used to determine the wax content. X-ray diffraction analysis was employed to determine the crystalline properties, including the crystallinity index and crystallite size. In addition, a Fourier-transform infrared (FTIR) spectroscopic study was also conducted to examine lignocellulosic fibers and determine their magnitudes of cellulosic composition. FTIR analysis is particularly useful in identifying the polymorphic structure of cellulose and in establishing the relationship between the OH bands and various components of biofibers such as cellulose, hemicellulose, pectin, and lignin, among others [38].

After being treated with KMnO_4_, the BP fiber’s cellulose weight increased from 58.5% of raw fiber to 59.4%. Hemicellulose declined to 21.8% after KMnO_4_ action, while the raw fiber possessed 40.13%. Lignin forms a connection between cellulose and hemicellulose polysaccharides. The fire-resistant behavior of lignin is a crucial quality to enforce for composites. Lignin was reduced by 13.5% after permanganate treatment, and the amount of wax weakened from 0.31% to 0.23% [32]. Table 2. compares the chemical analysis of different fibers [39,40]. The Chemical Tests Laboratory, SITRA, and Coimbatore provided the chemical analysis testing.

### 4.2. XRD Analysis

Upsurge in the crystalline unit was observed in the permanganate-treated fibers. Crystalline peak appeared at 23°, and a diffracted amorphous was spotted around 18^ᵒ^. Crystallinity index (CI) was computed to be 83.4%, with a crystallite size of 6.4 nm. CI for BP fibers is greater than *Furcraea foetida* (52.6%), *Napier grass fibers* (62.4%), *Nelumbo nucifera* (48%), *Sansevieria ehrenbergii* (52.27%), and *Acacia planifrons* (65.38%) [28,33]. The literature data show that higher variation in crystallinity index was registered for outer fibers than the inner core fibers of *bamboo plant* while subjected to alkali action. A good packing of cellulosic chains on a specific portion is responsible for this better crystalline output [41]. Surface morphology pictures of KMnO_4_-treated BP revealed more ordered packing of fibrils than raw BP.

**Figure 2:**
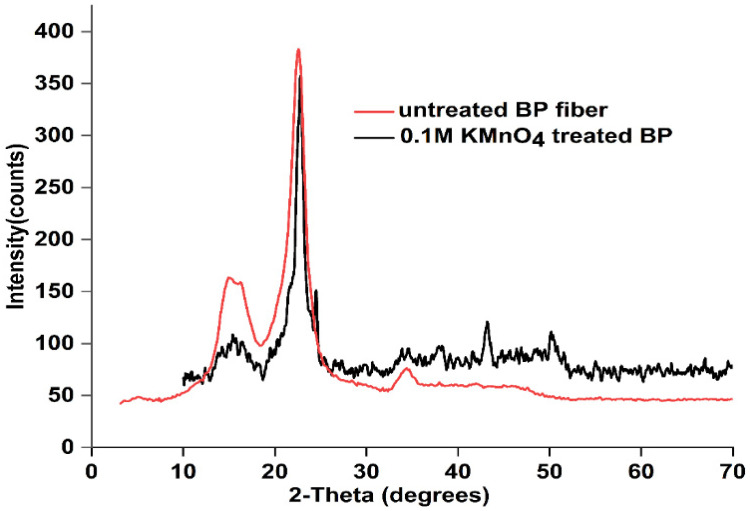
XRD graph of untreated and KMnO_4_-treated BP fiber.

Crystallite size of permanganate-treated fiber (6.4 nm) is bigger than *flax* (2.8 nm), *Thespesia populnea* (3.57 nm), and *cotton* (5.5 nm). The thermal characteristics are presumed to be improved by higher celluloses, larger CI, and CS because the intramolecular H-bond prevents thermal expansion, thereby imparting more stability to the fiber [42]. Smaller reaction sites reduce the surface area of crystallite, which increases the crystallite strength and durability, but conversely reduces the water absorption speed [43]. The merged XRD graph of raw and permanganate-treated BP is displayed in Figure 2, and the details on crystallinity are tabulated in Table 2.

### 4.3. Tensile Testing

Mechanical behavior of fibers is directly concerned with cellulose, as it gives sudden response to polymerization activities [44,45]. Tensile values of KMnO_4_-treated BP (198 MPa), followed by steam action for 15 min, was greater than raw BP fibers (92 MPa). Tensile modulus of treated BP is 4.40 GPa. Maximized values of fiber strength owes to the breakdown of amorphous entities housed within the fibrillar arrangement. Along with the exclusion of hemicellulose, lignin, wax, etc., from BP fiber, permanganate treatment rearranged the cellulosic chains by breaking and loosening the hydrogen network chain of the microfibrils [46]. Tensile values of treated BP are more than *Manicaria saccifera* (72.09 MPa), *Coir fibers* (95–174 MPa), and *ariel roots of Banyan fiber* and lower than *Cissus quadrangularis* (200.39 MPa) [47] and *Artichoke fibers* (201 MPa) [46,48,49]. Additionally, twisted single bundle fibers such as those from *Nelumbo nucifera* have higher tensile strength (427.7 MPa) than single fibers [43,44]. Tensile values of BP fiber are compared in Table 3 below.

Structural applications are adhered by the cellulose availability and microfibrillar arrangement. Cellulosic microfibrils in the fiber axis lead to elongation and creation of microfibrillar angle, which is straight-away connected to the tensile strength [51]. Ductility of plant fibers show an inverse relation with microfibrillar angle. The bigger the orientation angle with fiber axis is, the higher the probability of fibers to upset with wrecking [52]. After being treated with permanganate, microfibrillar angle (MFA) of Butea fiber was shrunk from (21.11 ± 14.08°) to (16.88 ± 9.87°), which is comparable to other fibers such as *Heteropogon contortus* (14.53 ± 0.53°) [47] and *Thespesia populnea* (13.94 ± 1.21°) [47].

### 4.4. Tg-Dta Analysis

**Figure 3:**
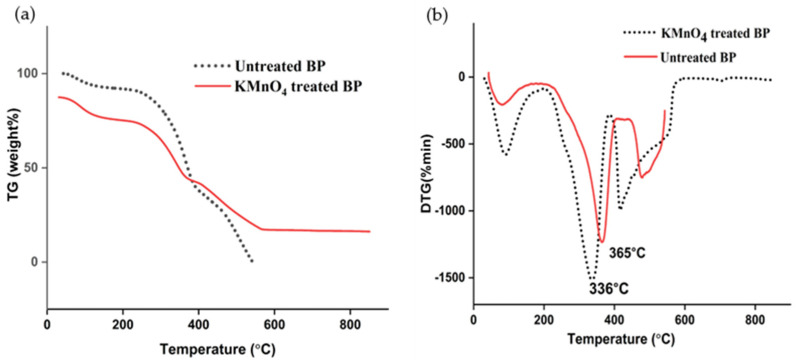
(**a**): TG curves of raw and treated BP; (**b**): DTG curves of raw and treated BP.

Fiber responses to various temperatures are studied using thermogram data. Tg-DTG curve is plotted in Figure 3a. Three-step degradation was seen in the permanganate-treated BP. Initial mass loss of 16% was noted between 50–200 °C, which is mainly due to the elimination of water. A profuse mass loss of 44% was recorded in the second stage of degradation up to 400 °C. Between 320–400 °C, cellulose exclusion has taken place, before which hemicellulose had expired between 200–320 °C. A final mass loss was stretched to 560 °C, focusing on the decomposition of lignin [18].

DTG curve revealed a significant disintegration peak at 336 °C, registering the removal of cellulose, closely matching the TG curve. The degradation peak for the raw BP was extended to 366 °C, higher than the KMnO_4_-treated fibers. Loss of lignin happens around 280–500 °C. Considerable loss of lignin nurtures thermal viability. However, chemical modification wiped out most lignin from the fiber arrangement leading to the earlier arrival of degradation temperature in the KMnO_4_-treated fiber than the raw BP. A comparison analysis showed that, among the fire retardant, rot retardant, water retardant, and raw jute fibers, raw fibers had the highest decomposition temperature [53]. In addition, incremented heat transfer to fibers would minimalize the melting heat, and it was noted to be lower in treated textile fiber than the untreated fiber [54]. These facts could have ignited the early decomposition in the permanganate-treated BP. The oxidation of hydrogen bonds between cellulose and hemicellulose caused by KMnO_4_ treatment has led to the formation of cellulose manganate complex [18]. This might have stretched the thermal stand by a temperature up to 240 °C. Similar degradation peaks were noticed in *jute* (365 °C), *sisal* (347 °C) and *buriti* (334 °C) [41].

#### 4.4.1. Differential Scanning Calorimetric Data

**Figure 4:**
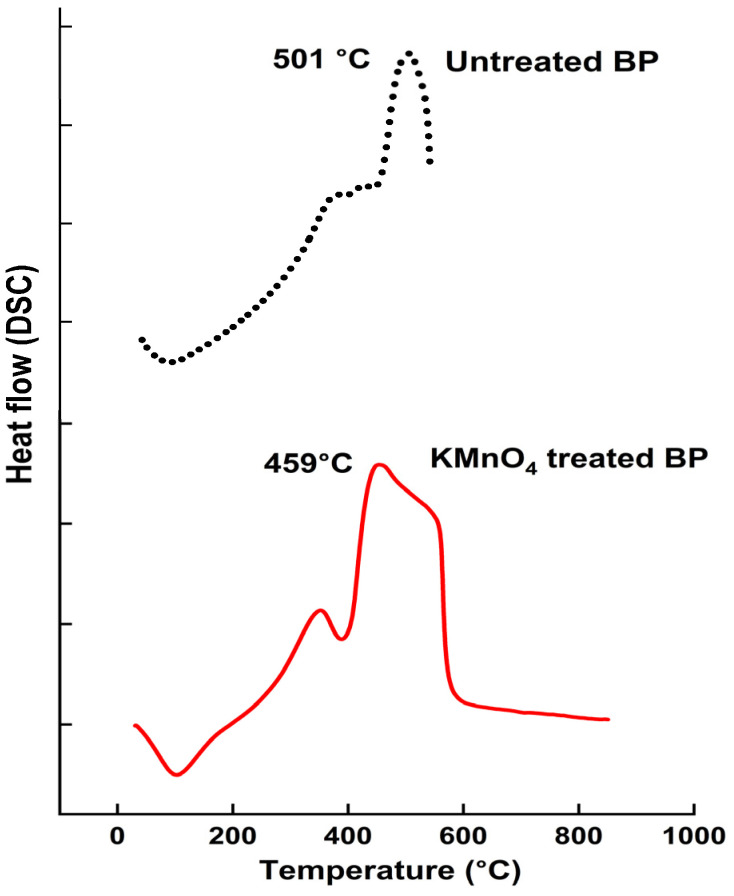
DSC curves of untreated and KMnO_4_ treated BP.

DSC profiles of untreated and KMnO_4_-treated BP fibers are portrayed in Figure 4. A wide range of components move out from BP at varying temperatures. Moisture is the chief entity that gets removed within the first level of heating, all over 100 °C. In the KMnO_4_ treated BP, heat absorption left a peak around 400 °C, indicating the exemption of cellulose. Lignin takes a wider degradation rate as it is composed of numerous O_2_ functional group structures with diverse heat stability [55]. An exothermic peak at 459 °C liberated the lignified compartment from the treated BP. However, raw BP showcased an extended exclusion of lignin around 501 °C. Thermal behavior of BP fibers is listed in Table 4.

#### 4.4.2. Activation Energy of BP

The activation energy of BP fibers was computed from Coasts–Redfern approximation [33].

(7) $$\log\left[\frac{-\log\left(1-\alpha \right)}{T^{2}}\right]=\log\frac{\mathrm{AR}}{\;\beta \mathrm{Ea}}\left[1-\frac{2\mathrm{RT}}{\mathrm{Ea}}\right]-\frac{\mathrm{Ea}}{2.303\mathrm{RT}}$$

**Figure 5:**
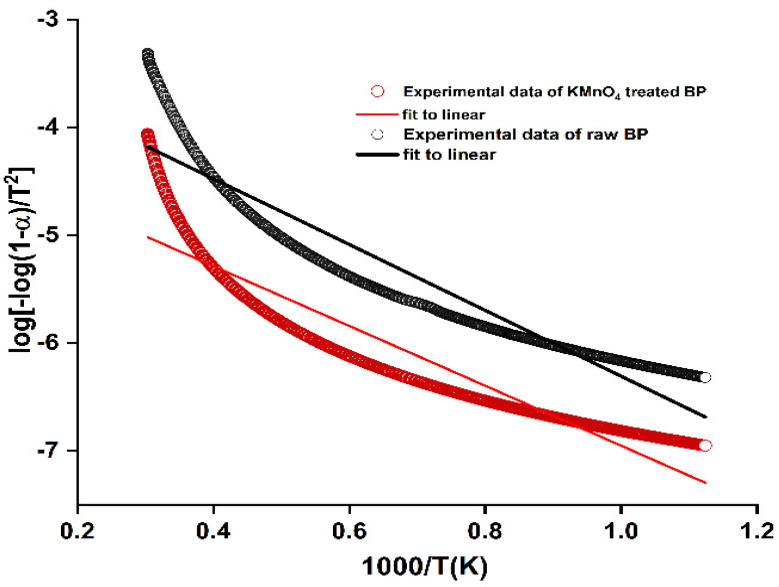
Activation energy plot of BP fibers.

A linear plot between log [−log(1 − α)/T^2^] and 1000/T estimated the activation energy Ea, which is the prime factor in fixing the thermal stability of reinforcement. Kinetic activation energy for the permanganate-treated fibers are 62.80−63.46 KJ/mol, which is lower than the untreated BP 73.15 KJ/mol. Thermal stability of biofibers are projected through their degradation temperatures. Higher bond energy results in improved thermal stability and activation energy [56]. Thermal stability is dominant in the raw BP, and so Ea of permanganate-treated BP is lower than the raw BP. Ea curve is plotted in Figure 5.

Higher activation energy levels hasten the deterioration of other thermal properties, and so it is crucial to maintain an optimum energy values to withhold the stability. Other fibers such as *Saharan Aloe vera cactus* (74.35 KJ/mol), *Prosopis julflora bark* (76.72 KJ/mol), and *Coccinia grandis* L. (73.43 KJ/mol) show similar kinetic energy values [57,58].

### 4.5. Thermal Conductivity

**Figure 6:**
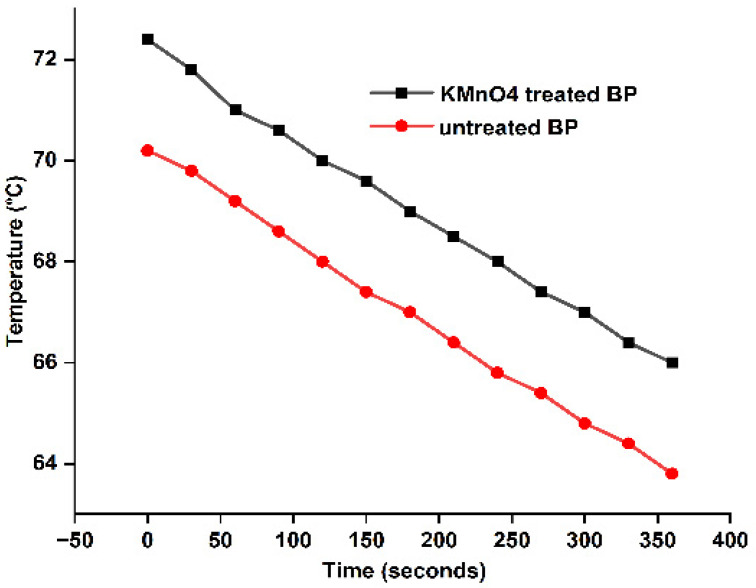
Thermal conductivity plot of raw and KMnO_4_-treated BP.

Fibers show differences in thermal conduction in varying surroundings. Although straw fiber imbibed with clay matrix improves insulation, the composites’ poor tensile strength limited its direct use in building applications [59], which neatly orients the role of conduction with the mechanical behavior. Lee’s disc setup was employed to study the insulation behavior of Butea fibers. Fiber thickness and porosity alter the conductivity. K values for raw and KMnO_4_-treated fibers are 0.029 W/mk and 0.032 W/mk, respectively. Lower thermal stability than the raw BP could explain the treated BP’s higher K value [56]. Permanganate-treated fibers gave higher Ea, which is compared with *Areca husk* fibers (0.021 W/mk) [60]. *Cotton stalk* fiber boards with heat conduction between 0.0585 and 0.0815 W/m K were created for ceiling applications [61]. Lowered K values provide a scope for utilizing Butea fibers for insulation applications when introduced in composites. Table 4. and Figure 6. give the linear action of thermal conductivity of raw and KMnO_4_-treated BP fiber.

### 4.6. Morphological Studies Using SEM

**Figure 7:**
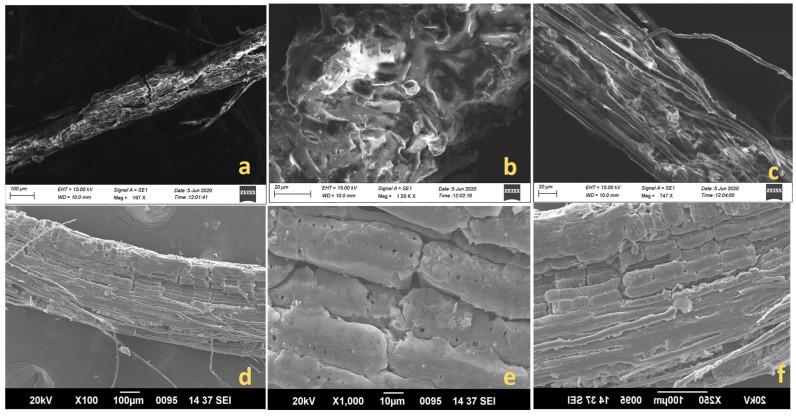
(**a**,**d**): SEM photographs of longitudinal sections of raw and treated BP; (**b**,**c**): SEM photographs of surface sections of raw BP; (**e**,**f**): SEM photographs of surface sections of potassium permanganate-treated BP.

Surface morphologies are diligent while considering fibers to be paired with matrix. Figure 7a–f. show the surface variations in raw and treated fibers, respectively. Raw fibers show projections on their surface, and fibrils are seen to be clouded with impurities, wax, hemicelluloses, and lignin. Microfibrils are unevenly oriented along the long axis. The presence of cellulose imparts a rough texture [62]. Figure 7b shows a flared structure in the raw BP, which could be easily distinguished from the ordered arrangements of treated BP. KMnO_4_ action on the fibers might have eroded the amorphous constituents by breaking the linkage between the cellulose and hemicelluloses [63]. However, micrographs of permanganate-treated BP display a shallow depression along the long axis [64,65]. Fine pores appearing on the surface could indicate a good bonding between the reinforcement and matrix for composite-making [6]. The longitudinal arrangement of cellulosic components in Figure 7d of treated BP was more aligned than the raw BP in Figure 7a. Surface sections of KMnO_4_-treated BP was nearly rod-shaped, which resulted in the dismissal of linking agents within the BP’s fiber space [42].

### 4.7. FTIR Analysis

**Figure 8:**
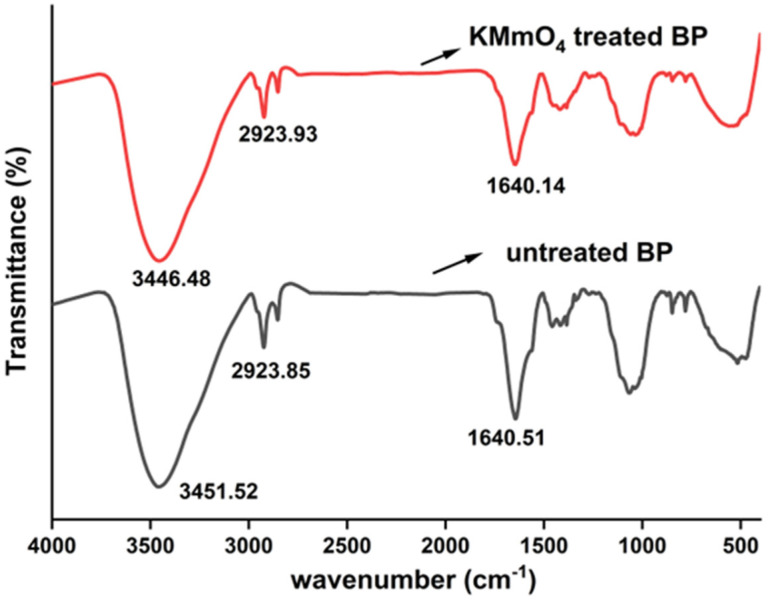
FTIR representation of raw and KMnO_4_ treated BP fiber.

Figure 8 spots the Fourier-transform infrared spectroscopic images of Butea parviflora. Vibrations due to amorphous groups are comparatively disappeared in the treated fiber. Wide and strong peaks witnessed within the limits 3450−3350 cm^−1^ are due to the disturbances executed by the hydrogen bonded OH group of the fiber [66,67,68] Absence of minor peaks witnessed in 1384 cm^−1^ might be due to the oxidation of lignin as a consequence of permanganate action [18].

A peak rise in the area 646.78 cm^−1^ is involved in CS stretching [28]. Specific minor variations in peaks are observed for permanganate-treated BP, since several functional groups of amorphous constituents had removed during KMnO_4_ action. Vibrations differing for untreated and permanganate-treated BP are spotted in Table 5 [69,70,71,72].

## 5. Conclusions

Potassium permanganate-treated Butea parviflora (BP) fiber meets essential requirements for reinforcements. BP fibers have better physical and chemical behavior compared to most other bio fibers in the literature. BP fibers have low density (1.40 g/cc), making them suitable for lightweight composite applications.

Chemical analysis of permanganated BP showed a higher cellulose percentage (59.4%) and lowered hemicellulose content (21.8%). Semicrystalline fibers treated with permanganate showed an increase in the cellulosic CI and CS of 83.47% and 6.4 nm, respectively, resulting in better crystalline outcome, an essential behavior for bio-composites.

Tensile strength of fibers treated with KMnO_4_ increased to 198 MPa from 92 MPa (raw BP), and a tensile modulus of 4.40 GPa was observed for treated BP. Microfibrillar angle in treated BP was reduced (16.88 ± 9.87°) compared to raw fiber (21.11 ± 14.08°), indicating improved strength performance of BP composites. DTG curve for KMnO_4_-treated and untreated fibers, respectively, showed a maximum degradation peak at 336 °C and 366 °C. Raw BP’s thermal behavior was slightly superior to that of the treated BP.

Treating fibers with 0.1 M permanganate did not significantly increase the degradation temperature. Activation energy for the treated fiber was between 62.80–63.46 KJ/mol, comparatively lower than raw BP, indicating improved stability.

Thermal conductivity (K) was approximately 0.032 W/mk, which could be altered for insulation applications.

SEM photographs revealed a challenging variation on the fiber surface, indicating excellent adhesion with the matrix. Spectroscopic vibrations of IR studies marked a minor variation in the various functional groups of cellular components.

Comprehensive analysis of all the above activities of BP fiber reveals a good standard for introducing this permanganate-treated Butea parviflora for making eco-friendly composites. This study highlights the potential of utilizing BP fiber in composite materials due to its low density, improved tensile strength, and remarkable crystalline behavior. Further research is planned to explore the benefits of surface modifications of Butea fibers and to develop BP fiber composites that maximize its properties.

In conclusion, the results of the study demonstrate that potassium permanganate-treated Butea parviflora fiber has the potential to serve as a suitable reinforcement material for biodegradable composites. The physical and chemical properties of the fiber, including its low density, high cellulose percentage, and improved crystalline outcome, make it a promising candidate for lightweight and strong biocomposites. The SEM photographs also indicate that the fiber surface has desirable variations for excellent adhesion with the matrix, further enhancing its potential as a reinforcement material. Moreover, the biodegradability of the BP fiber makes it an environmentally friendly option for developing sustainable and eco-friendly composites. Overall, this study highlights the potential of permanganate-treated BP fiber for developing biodegradable composites that have promising mechanical and thermal properties.

## Figures and Tables

**Table 2:** Details on chemical constituents, crystalline behavior of fibers.

Fibers	Chemical Composition (wt %)					Crystalline Properties	
	Cellulose	Hemicellulose	Lignin	Moisture	Wax	Crystallinity Index	Crystallite Size
Raw BP	58.5	40.13	18.09	11.63	0.31	83.63	7.5 nm
KMnO_4_-treated BP	59.4	21.8	13.5	13.53	0.23	83.47	6.4 nm
flax	64.10	16.70	2.0	10	1.5	–	–
*Sansevieria cylindrica*	79.7	10.13	3.8	–	0.09	60.0	86
*Cyperus pangorei*	68.5	–	17.8	9.19	0.17	41.0	–
*Ariel roots of banyan*	67.63	13.46	15.62	10.21	0.81	72.47	6.28

**Table 3:** Assessment of tensile behavior of Butea parviflora (BP) over other fibers.

Fibers	Tensile Strength (MPa)	Tensile Modulus (GPa)	Elongation at Break (%)	Reference
Raw BP	92.64	2.16	7.2	Present work
KMnO_4_-treated BP	198.12	4.40	4.5	Present work
*Cissus quadrangularis*	200.39	4.89	3.57–8.37	[31]
*Manicaria saccifera*	72.09	2.20	–	[50]
*Ariel roots of banyan fiber*	19.37	1.8	1.8 ± 0.40	[30]
*Cyperus pangorei*	196	11.6	1.69	[40]

**Table 4:** Thermal and (Ea) details of Butea parviflora (BP).

Samples	Max Degradation Temperature (°C)	Activation Energy (E_a_)	Thermal Conductivity (K)
Untreated BP	365	73.15 KJ/mol	0.029 W/mk
KMnO_4_-treated BP	336	62.80−63.46 KJ/mol	0.032 W/mk

**Table 5:** Spectroscopic assignments of Butea parviflora (BP).

Wavenumber (cm) ^−1^		Vibrational Band Assignments
Raw BP	0.1 M KMnO_4_ Treated BP	
3451.52	3446.48	H_2_ bonded O–H group of cellulose
2923.85	2923.93	C–H_2_ stretching of cellulose
2853.34	2852.31	C–H stretching of hemicelluloses
1640.51	1640.14	C=O stretching of acetyl group in hemicellulose
**–**	1423	C–H_2_ symmetric bending in cellulose
1384.21	–	Asymmetric COC stretching of lignin
**–**	1269.41	C-O stretching of acetyl group in lignin and hemicelluloses
1068.81	–	C-O stretching variations of polysaccharides
1036.97	1034.82	C–O stretching
873.40	877.24	β-glycosidic linkage in monosaccharides
780.78	780.02	CO stretching
**–**	646.78	CS stretching
517.10	–	Out of plane bending of OH

## Data Availability

Available on Request.
